# LEDGF/p75 controls 3D-localization of HIV-1 provirus in the nuclear compartment of infected cells

**DOI:** 10.1186/1742-4690-10-S1-O10

**Published:** 2013-09-19

**Authors:** Annegret Boll, Lenard Vranckx, Cristina Di Primio, Valentina Quercioli, Rik Gijsbers, Zeger Debyser, Anna Cereseto

**Affiliations:** 1 Centre for Integrative Biology (CIBIO), University of Trento, Mattarello, Italy; 2 Centre for Molecular Medicine, Department of Pharmaceutical and Pharmacological Sciences, KU Leuven, Leuven, Belgium; 3 Laboratory of Neurobiology, Scuola Normale Superiore, Pisa, 56124, Italy

## Background

Integration of HIV-1 provirus occurs mainly in transcriptionally active units. Furthermore HIV-1 PICs preferentially localize in decondensed chromatin regions near the nuclear rim [1]. A technique recently developed in our lab, Single Cell Imaging of HIV-1 Provirus (SCIP), allowed us to determine that proviral DNA also localizes near the nuclear rim [2]. Emerging evidence suggests that nuclear functions such as transcription, DNA repair and replication are coordinated by a specific nuclear dynamic architecture and not just by the linear sequence of the nucleotides. Similarly, HIV-1 nuclear events might be controlled by the organization of the nuclear compartment. The aim of this study is to analyze whether LEDGF/p75 plays an important role in 3D-localization of HIV-1 in the nuclear volume of infected cells.

## Materials and methods

SCIP allows to visualize the viral genome at the level of single nuclei of individual cells. Compared to conventional techniques SCIP offers the possibility to analyze the topology of infected cells and thus the localization of proviral DNA in the nuclei. For details see Di Primio *et al*. [2].

## Results

Here we used SCIP [2] to investigate the role of LEDGF/p75 on the 3D-localization of integrated provirus in the nuclei of HIV-1 infected cells. We found that the localization of proviral DNA in the nucleus of an infected cell depends on LEDGF/p75 expression. Integrated HIV-1 genomes localize near the nuclear rim in HIV-1 infected cells, whereas nuclei of LEDGF/p75 knockdown cells show a more random distribution of proviral DNA. Finally we proved that retargeting of integration sites in CBX-LEDGF cells [3] coincides with 3D-randomization of proviral DNA.

## Conclusion

Using SCIP we demonstrated that HIV-1 provirus integrates preferentially close to the nuclear periphery, where also PICs are visualized [2]. In the absence of LEDGF/p75 or in cells expressing a LEDGF/p75 hybrid the integration spots acquire a random 3D distribution within the nucleus indicating a clear role of LEDGF/p75 in spatial localization of HIV-1 in the nuclei of infected cells. Therefore, these results prove that LEDGF/p75 in addition to linear tethering is responsible for 3D-tethering of the virus. This conclusion is further supported by evidence showing that forced retargeting of HIV-1 towards heterochromatin regions [3] results in random distribution in the nuclear compartment.
